# Good Advice

**Published:** 1855-01

**Authors:** 


### GOOD ADVICE.

The following "Advice to Housewives' contains some useful hints:

Britannia should be first rubbed gently with a woolen cloth and sweet oil, then washed in warm suds, and rubbed with soft leather and whiting. Thus treated, it will retain its beauty to the last.

New iron should be gradually heated at first; after it has become inured to the heat, it is not likely to crack.

It is a good plan to put new earthenware into water, and let it heat gradually until it boils—then cool again. Brown earthenware, particularly, may be toughened in this way.—A handful of rye or wheat bran, thrown in while it is boiling will preserve the glazing so that it will not be destroyed by acid or salt.

Clean a brass kettle, before using it for cooking, with salt and vinegar.

The oftener carpets are shaken, the longer they will wear. The dirt that collects under them grinds out the threads.

If you wish to preserve fine teeth, always clean them thoroughly after you have eaten your last meal at night.

Woolen should be washed in very hot suds, and not rinsed. Lukewarm water shrinks woolen goods.

Do not wrap knives and forks in woolens. Wrap them in good strong paper. Steel is injured by lying in woolens.

Barley straw is best for beds; but dry corn husks slit into shreds are better than straw.

When molasses is used in cooking, it is a great improvement to boil and skim it before you use it. It takes out the unpleasant raw taste, and makes it almost as good as sugar.

When molasses is used much for cooking, it is well to prepare one or two gallons at a time.

Never allow ashes to be taken up in wood or put into wood.

Always have your matches and lamp ready for use in case of sudden alarm.

Have important papers all together, where you can lay your hands upon them at once in case of fire.

Use hard soap to wash your clothes, and soft to wash the floors. Soft soap is so slippery that it wastes a good deal in washing clothes.

